# In Vitro and In Situ Characterization of Psychrotrophic Spoilage Bacteria Recovered from Chilled Chicken

**DOI:** 10.3390/foods12010095

**Published:** 2022-12-24

**Authors:** Xinxia Wang, Zaitian Wang, Zhilan Sun, Daoying Wang, Fang Liu, Lin Lin

**Affiliations:** 1 School of Food and Biological Engineering, Jiangsu University, Zhenjiang 212013, China; 2 Jiangsu Key Laboratory for Food Quality and Safety-State Key Laboratory Cultivation Base, Ministry of Science and Technology, Nanjing 210014, China; 3 Institute of Agricultural Products Processing, Jiangsu Academy of Agricultural Sciences, Nanjing 210014, China; 4 Key Laboratory of Cold Chain Logistics Technology for Agro-Product, Ministry of Agriculture and Rural Affairs, Nanjing 210014, China

**Keywords:** chilled chicken, spoilage bacteria, *Pseudomonas*, proteolysis, volatile odor

## Abstract

Spoilage bacteria play a remarkable role in the spoilage of chilled chicken. In this paper, a total of 42 isolates belonging to 16 species of four genera were isolated from chilled chicken and displayed different characterizations of psychrotrophic spoilage. Six isolates of J7, J8, Q20, Q23, R1, and R9 with differences in proteolytic capabilities were further characterized for in situ spoilage potential evaluation. *Pseudomonas lundensis* J8 exhibited the strongest spoilage potential in situ, displaying a fast growth rate, increased pH velocity, high total volatile basic nitrogen, and high peptide content in the chicken samples. The volatile flavor analysis of chicken samples via electronic nose indicated that the content of characteristic odors representing spoilage, including sulfides, organic sulfide, and hydride, increased during storage. Additionally, the principle component and correlation analyses revealed that the spoilage odors produced by different species of bacteria were significantly different and positively correlated with the results of protease activity in vitro. The characteristics of spoilage bacteria in chilled chicken provided a comprehensive insight into microbial assessment during storage.

## 1. Introduction

As one of the most valuable livestock products, chicken contains abundant protein and minimal fat, which satisfies the rising demand for protein [1,2]. However, fresh chicken is perishable, and thus chilled chicken has gradually become the primary vending module in poultry markets to reduce the occurrence of chicken spoilage [3,4]. Hence, chilled chicken quality has gained increasing interest in consideration of consumer concerns. With the increase in storage time, the freshness of chilled chicken decreases, resulting in quality deterioration [5]. In addition to endogenous enzymes and lipid oxidation, microbial contamination is the main reason for chicken spoilage [6,7]. Consequently, numerous studies have focused on spoilage bacteria identification in chicken. Numerous bacteria have been identified as microorganisms responsible for chilled chicken spoilage, including *Pseudomonas*, *Shewanella*, *Listeria*, and *Brochothrix* [1,8,9]. Many studies have focused on the variety and abundance of bacteria on chicken while ignoring the examination of spoilage bacteria on chilled chicken with strong catabolic capabilities [10]. Therefore, an analysis of the characteristics of spoilage strains based on pure incubation is necessary to effectively understand chilled chicken spoilage.

Psychrophilic aerobic bacteria such as *Pseudomonas* are the most predominant causes of chilled meat spoilage [11]. *Pseudomonas* strains have the ability to hydrolyze protein and lipids, which is commonly associated with meat spoilage [12,13]. In addition, *Pseudomonas* can form biofilms to promote its growth and resist adverse growing conditions, which increases the possibility of meat spoilage [14,15]. Therefore, many researchers have devoted themselves to studying the *Pseudomonas* group in spoiled meat. Many studies have indicated that *Pseudomonas fluorescens*, *Pseudomonas fragi*, *Pseudomonas lundensis*, and *Pseudomonas putida* are closely associated with chilled meat spoilage, whereas the capability of *Pseudomonas* species to decompose poultry is different [14,15,16,17]. For example, Zhang et al. found that the intestinal adhesion ability of *P. putida* was the pivotal factor in the spoilage degree of chilled fish by accessing the spoilage ability of four *P. putida* strains isolated from spoiled tilapia [18]. Papadopoulou et al. indicated that *P. fragi* displayed the highest spoilage capacity on pork meat by comparing it with *P. putida*, *Leuconostoc mesenteroides*, and *Lactobacillus sakei* [19]. Similarly, Wang et al. evaluated the in situ spoilage potential of *P. fragi* and *P. fluorescence* at refrigeration temperature and found that *P. fragi* exhibited the strongest spoilage potential [20]. Nevertheless, as one of the most important putrefying bacteria that cause chilled chicken spoilage, the in situ catabolic characteristics of *P. lundensis* on chilled chicken have seldom been reported. Furthermore, in addition to *Pseudomonas*, the absolute dominant spoilage bacteria, other dominant spoilage bacteria such as *Aeromonas* and *Brochothrix* are also found on the surface of naturally spoiled chicken. Since previous studies indicated that *Aeromonas* are the dominant pathogen resulting in fish spoilage, its characterizations in chilled chicken spoilage were rarely well-reported [21]. Recently, Shao et al. characterized the spoilage heterogeneity of 12 *Aeromonas* strains isolated from chilled chicken and found that *Aeromonas salmonicida* 29 demonstrated the strongest spoilage potential [22]. Additionally, Zhang et al. indicated that *Brochothrix* is also one of the dominant spoilage bacteria in chilled chicken based on microbiome and metabolomics. However, the microbiota on chicken samples is complex, wherein symbiosis antagonism, competition, and other microbial interactions may lead to changes in chicken spoilage characteristics [1,23]. Therefore, analyzing the specific spoilage characteristics of a single bacterium is necessary.

The purpose of the present study was to reveal the potential bacteria associated with chilled chicken spoilage. Skim milk agar was used in this study to prescreen the spoilage potential of 42 bacterial isolates from chilled chicken, which belonged to 16 species of four genera. The strains with significantly different spoilage capability in vitro were then screened for in situ spoilage potential evaluation. Quality changes were evaluated considering microbial colony count, pH, TVB-N, and peptide content along with the use of an E-nose to determine the specific spoilage characteristics of *Pseudomonas lundensis* J7, *Pseudomonas lundensis* J8, *Aeromonas hydrophila* Q20, *Aeromonas media* Q23, *Brochothrix thermosphacta* R1, and *Brochothrix thermosphacta* R9. These findings will lead to an effective understanding of the spoilage characteristics of different microbial strains, which will provide a primary basis for enhancing the quality of chilled chicken.

## 2. Materials and Methods

### 2.1. Bacterial Species

A total of 42 strains previously isolated from chilled chicken were provided by the Jiangsu Academy of Agricultural Sciences (Nanjing, China). Table 1 shows that these isolates belonged to different taxonomic groups, including *Pseudomonas* spp. (*P. fluorescens*, *P. lundensis*, and *Pseudomonas psychropila*), *Aeromonas* spp. (*A. hydrophila*, *A. media*, and *Aeromonas salmonicida*), *Brochothrix* spp. (*B. thermosphacta*), *Psychrobacter pulmonis*, *Cronobacter sakazakii*, and *Escherichia coli*.

### 2.2. Evaluation of Extracellular Protease Activity

According to the reported literature, proteolytic activity in vitro was measured using a skim milk medium [24,25]. The skim milk agar was prepared by adding 2% (*w*/*v*) skim milk into plate count agar (PCA). First, all the strains were cultured in Luria–Bertani (LB) broth for 24 h at 28 °C with shaking at 200 rpm. Next, the optical density of cells at 600 nm (OD_600_) was adjusted to around 0.40. Then, 2 μL bacterial dilution was dotted onto skim milk agar and incubated at 28 °C for three days. Subsequently, bacterial colony diameter (BCD) and protein decomposition zone diameter (DZD) were measured via a Vernier caliper (0.01 mm precision). The proteolytic activity was accessed by the ratio of DZD to BCD (DZD/BCD) according to the literature [25]. Strains with the weakest and strongest proteolytic activities in each bacterial genus were selected to assess the in situ spoilage potential. Thus, the strains *P. lundensis* J7, *P. lundensis* J8, *Aeromonas hydrophila* Q20, *Aeromonas media* Q23, *B. thermosphacta* R1, and *B. thermosphacta* R9 were selected for spoilage potential evaluation.

### 2.3. Spoilage Potential Evaluation

#### 2.3.1. Strain Culture and Inoculation

First, strains stored in glycerol (25%, *v*/*v*) at −80 °C were activated in 5 mL LB broth at 28 °C for 12 h with 200 rpm. Then, a 1% (*v*/*v*) inoculation of activated strains was transferred to 10 mL LB broth and cultured to the late logarithmic stage (OD_600_ = 1.0) under the same conditions, and approximately 10^8^ CFU mL^−1^ was achieved. Afterward, the culture of different strains was individually diluted in 0.85% sterilized NaCl (ready for use) to prepare the bacterial inoculum. Chicken breasts purchased from Suguo supermarket were sterilized by irradiation (6 KGy, Nanjing Joy Irradiation Technology Co., LTD., Nanjing, China) as described by Wang et al. [26]. The aseptic chicken breasts were chopped into muddy flesh at a super-clean workbench before artificial contamination. Subsequently, the inoculum of each strain prepared above was inoculated into muddy flesh samples (10 g) at a concentration of 10^4^ CFU g^−1^. Uncontaminated muddy flesh served as control. To simulate the storage conditions of chilled chicken, all samples were stored at 10 °C for three days. The microbial total viable count (TVC) and pH were determined daily. In addition, total volatile basic nitrogen (TVB-N), polypeptide content, and odor analysis were performed on the last day of storage.

#### 2.3.2. Microbial Analysis

The bacterial TVC of samples was measured daily. Individually, samples (10 g) were aseptically transferred into homogeneous bags containing 90 mL 0.85% sterile normal saline, which was homogenized for 5 min at room temperature. Thus, the bacteria on the samples were diluted tenfold. Then, 0.5 mL homogenized solution was serially diluted in 4.5 mL 0.85% sterile NaCl. Subsequently, 100 μL dilution with appropriate concentration was spread on the selective culture medium of corresponding bacteria (*Pseudomonas*—*Pseudomonas* CFC selective medium, *Aeromonas*—*Aeromonas* selective medium, *Brochothrix*—STAA medium, Qingdao Haibo Biotechnology Co., LTD, Qingdao, China), and the diluent of control was coated on PCA. After coating, the medium agar plates were cultured upside down in an incubator at 28 °C for one day. The amounts of bacteria in the chicken breast were expressed as colony-forming units in each gram sample (CFU g^−1^).

#### 2.3.3. pH and Total Volatile Basic Nitrogen (TVB-N) Analysis

pH was determined in accordance with the method proposed by Hwanhlem et al., with minor modifications [27]. In brief, a 10 g muddy flesh sample was homogenized with 90 mL ultrapure water. Three aliquots randomly taken from the sample solution were measured via an electronic pH meter.

TVB-N was determined in accordance with the Conway microdiffusion method included in the determination of total volatile basic nitrogen in food (GB5009.228-2016). Briefly, 10 g muddy flesh was transferred into a 100 mL centrifuge tube with 40 mL pure water and homogenized by a high-speed homogenizer for 30 s, followed by centrifuging under 5000 rpm for 2 min. Next, the supernatant was filtered by qualitative filter paper, and the filtrate was used for the determination of TVB-N. The results were converted to mg per 100 g of samples.

#### 2.3.4. Polypeptide Content Assessment

Polypeptide content was determined by the biuret method [28]. A total of 10 g of sample was mixed with sterilized pure water, and 5 mL sample solution was then put into a 10 mL centrifuge tube and placed in a boiling water bath for 15 min. After cooling to room temperature, the samples were centrifuged at 4000 rpm for 10 min and the supernatant was removed. Afterward, 10% (*w*/*v*) trichloroacetic acid (TCA) was used to precipitate the protein remaining in the supernatant. After reacting for 15 min, the solution was centrifuged at the same conditions above. Subsequently, 1 mL supernatant was evenly mixed with 4 mL biuret solution and reacted at 37 °C for 30 min; its absorbance was measured at 540 nm. Finally, the polypeptide content of the meat samples was calculated by the standard curve drawn using bovine serum albumin as standard. The calculation formula was as follows:

$$polypeptide\;content=\frac{absorance\;value-0.0702}{0.0278}\times dilution\;ratio,$$

where polypeptide content was expressed as mg g^−1^.

#### 2.3.5. Electronic Nose Measurement

The odor composition of chicken breast samples was analyzed using a PEN3 electronic nose system (Airsense, Germany) based on the method of Yin et al. [29]. The sensitive substances of 10 metal oxide gas sensors in the system are as follows: W1C: aromatic hydrocarbon; W5S: oxynitride; W3C: ammonia, aromatic molecule; W6S: hydride; W5C: olefin, aromatic, and memory; W1S: alkanes; W1W: sulfide; W2S: alcohols, some aromatic compounds; W2W: aromatic compounds, organic sulfides; W3S: alkanes and aliphatic groups. A total of 4 g of minced chicken breast samples were divided into a 20 mL headspace sample vial and sealed. The vials were placed into the tray for testing after the e-nose detector was calibrated. The headspace temperature was heated to 40 °C, and the chamber flow was set as 200 mL/min during the measurement. The measurement time was set at 180 s to obtain stabilized data. The cleaning time was 90 s to distinguish samples effectively. The samples were measured with 200 μL injection flow. Each treatment measured by e-nose comprised triplicate samples.

### 2.4. Correlation Analysis

To analyze the relationship between in vitro proteolytic capacity and in situ spoilage capacity intuitively, the correlation between in vitro proteolytic capacity and in situ spoilage characteristics at the last day of storage at 10 °C, including TVC, TVB-N, peptide content, and E-nose sensor responses, was investigated by correlation analysis. Particularly, the heatmap constructed on the basis of Pearson correlation coefficient facilitated the intuitive analysis, linking these spoilage characteristics together, which is helpful for further examination.

### 2.5. Statistical Analysis

All experiments in this work were performed in triplicate. The experimental results are expressed as the mean ± standard deviation. Differences between control and experimental groups were evaluated by one-way ANOVA using the SPSS statistics program (version 26.0, Chicago, IL, USA). The Tukey test (*p* < 0.05) was applied in all experimental indexes to identify whether the experimental results were of statistical significance. All the experimental results were plotted using Origin Pro 2021.

## 3. Results and Discussion

### 3.1. Proteolytic Activity Screening

**Figure 1:**
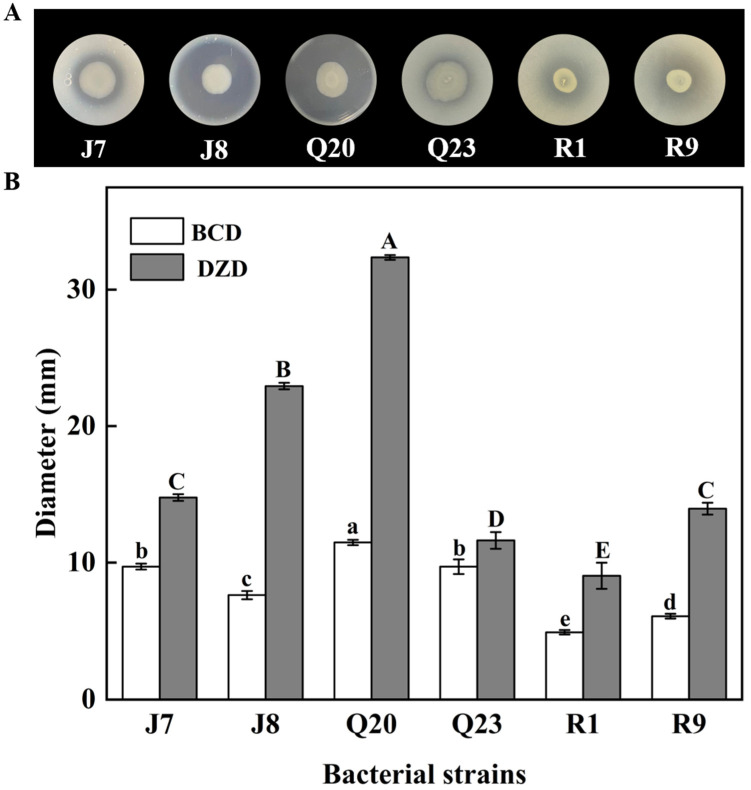
Proteolytic circle on skim milk agar (**A**), DZDs and BCDs (**B**) of *P. lundensis* J7, *P. lundensis* J8, *A. hydrophila* Q20, *A. media* Q23, *B. thermosphacta* R1, and *B. thermosphacta* R9. Different capital and lowercase letters represent significant differences of DZDs and BCDs (*p* < 0.05).

In the evaluation of in vitro proteolytic activity, protein degradation was used as a spoilage indicator to assess the spoilage potential of bacteria. According to the in vitro proteolytic assay, different microbial species indicated different proteolytic activities, and the proteolytic capability of different strains of the same species was also different [25]. As shown in Table 1, all the spoilage bacteria species isolated from chilled chicken in our laboratory exhibited different proteolytic activities. As shown in Figure 1, six isolates of J7, J8, Q20, Q23, R1, and R9 with differences in proteolytic capabilities were screened out according to the results in Table 1. Significantly, the DZD/BCD value of *P. lundensis* was the largest, followed by *A. hydrophila*, which exhibited strong in vitro proteolytic capacity. Previous studies indicated that psychrotrophic *Pseudomonas* species have been identified as the predominant spoilage microbes in chilled chicken during aerobic storage [30,31]. Aside from *P. fragi* and *P. fluorescens*, the most commonly identified species, *P. lundensis*, is also one of the most abundant *Pseudomonas* contributing to the spoilage of chilled meat [31,32]. Simultaneously, it is worth noting that other pathogenic bacteria, *Aeromonas* spp. and *Brochothrix* spp., are also relatively abundant on chilled chicken due to cross-contamination during food processing from causes such as inadequate cutting, unclean slaughter conditions, and contaminated water [33,34]. The proteolytic activity of *P. fragi* in chicken has been reported in numerous studies, while information regarding that of *P. lundensis*, *A. hydrophila, A. media*, and *B. thermosphacta* is limited [35,36].

### 3.2. Growth of Bacterial Strains in Meat

**Figure 2:**
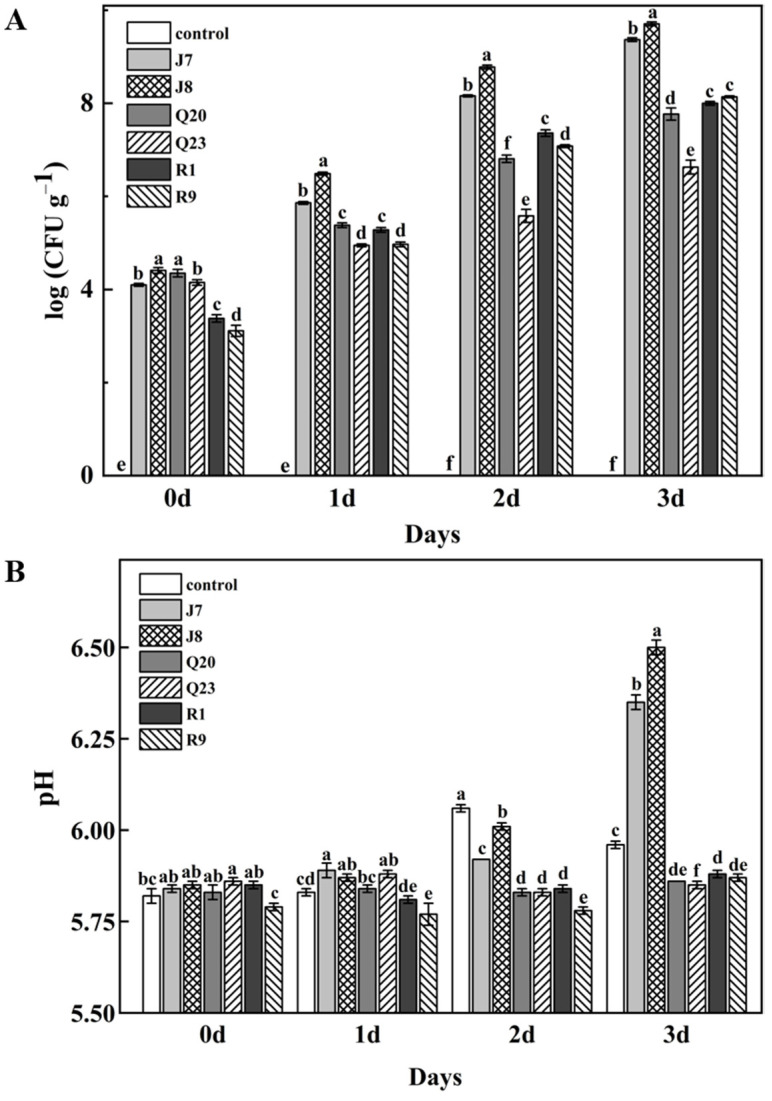
Microbiological growth (**A**) and pH changes (**B**) of chicken breast samples inoculated with *P. lundensis* J7, *P. lundensis* J8, *A. hydrophila* Q20, *A. media* Q23, *B. thermosphacta* R1, and *B. thermosphacta* R9 during storage at 10 °C for three days. Different lowercase letters denote significant differences at different times during storage (*p* < 0.05).

The growth of the six selected strains was determined daily during storage at 10 °C, and the result is shown in Figure 2A. During storage after inoculation, the growth rate of *P. lundensis* was the fastest and reached approximately 6 log CFU g^−1^ on the first day, which was already in the spoilage stage [22]. Moreover, the growth rate of *B. thermosphacta* R1 and R9 was second only to *P. lundensis* J7; *A. hydrophila* Q20 and *A. media* Q23 were the slowest. On the last day of storage, the TVC of *P. lundensis*, *A. hydrophila*, *A. media*, and *B. thermosphacta* all exceeded the bacterial limit of GB a16869-2005, indicating that all the chicken samples were rotten. Additionally, the TVC for each spoilage bacteria was different, the TVC of *Pseudomonas* especially was significantly higher than other bacterial species. Chilled storage is the most common method to store poultry meat, which can enforce a stressful environment condition for bacteria strains; the bacteria that can withstand stressful pressure will grow robustly and become dominant [37]. Additionally, the TVC of the control samples was 0 log CFU g^−1^ during storage, indicating that irradiation can effectively inactivate the microorganisms in chicken breasts [20]. 

### 3.3. pH Changes

As depicted in Figure 2B, the pH values of chicken samples were altered during storage. Initially, the pH values of all samples were approximately 5.80, which corresponded to previous studies in which the primary pH of chicken breast was 5.7–6.0 [38,39]. The pH values of chicken contaminated with *P. lundensis* J7 and J8 rapidly increased during storage, individually reaching 6.34 and 6.50 on the third day, whereas the pH values of other samples were almost lower than 6.0. The reasons for pH changes of meat are various, such as active glycolysis, storage temperature, and proteolytic degradation [40,41]. The optimum pH for growth and protease production of many *Pseudomonas* spp. is 6.0–8.0 [42]. Previous studies demonstrated that the by-products produced by *Pseudomonas* will contribute to the increased pH of meats, which is conducive to accelerating the rapid growth of *Pseudomonas* spp., followed by protein breakdown and nutrient loss, which eventually leads to accelerated spoilage [20,43].

### 3.4. TVB-N Results

**Figure 3:**
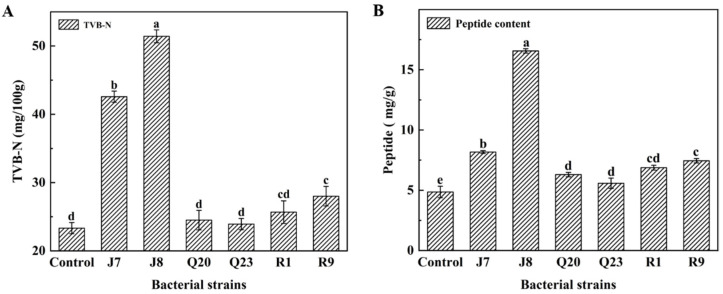
Peptide content (**A**) TVB-N (**B**) results at the end of storage of chicken breast samples inoculated with *P. lundensis* J7, *P. lundensis* J8, *A. hydrophila* Q20, *A. media* Q23, *B. thermosphacta* R1, and *B. thermosphacta* R9. Different lowercase letters indicate a significant difference among the samples contaminated with different bacteria for the same index.

TVB-N is the alkaline nitrogen-containing substance produced by protein decomposition, which reflects the spoilage level of meat [44]. Thus, TVB-N is frequently used as a measuring indicator of meat quality. As exhibited in Figure 3A, the TVB-N of different samples was evaluated at the end of storage. The TVB-N of samples individually incubated with *P. lundensis* J7 and J8 was higher than 40 mg/100 g, and *P. lundensis* J8 showed the strongest TVB-N generation capability, which is consistent with the final pH of the chicken samples. *P. lundensis* J8 revealed the most serious spoilage. Combined with previous studies, the *Pseudomonas* group is the bacterial genus that most potentially causes spoilage in chilled meat [31]. Similarly, Jia et al. found that the final TVB-N produced by *P. psychrophila* in chilled silver carp (*Hypophthalmichthys molitris*) reached a value of 50.80 mg/100 g, which was far more than *A. allosaccharophila* and *S. putrefaciens* [45]. According to the proteolytic analysis, *A. hydrophila* Q20 also exhibited strong proteolytic activity, while its growth state was worst and pH changes were nonsignificant, which is likely to be the main reason for its low TVB-N [20]. *B. thermosphacta*, which also rapidly grew on meat, showed moderate protease activity in the in vitro protease activity test. The initial TVC of *B. thermosphacta* R1 and R9 was small but their growth rates were fast. Thus, the final TVC of *B. thermosphacta* was slightly more than *Aeromonas* spp., which may be the reason that the TVB-N of *B. thermosphacta* was slightly greater than *Aeromonas* groups.

### 3.5. Proteolysis Extent Assay

As a principal component of meat, protein degradation could directly cause meat nutrient loss and sensory deterioration, which eventually lead to meat spoilage [20]. In particular, microbial proteinases significantly contribute to the hydrolysis of meat proteins. Many research works have studied the differences in proteolysis of spoilage bacteria in vitro using SDS-PAGE analysis, but information regarding the hydrolysis of proteins in situ is limited [24,46]. In the evaluation of in situ spoilage properties presented herein, the proteolytic activity of different spoilage bacteria was compared through the determination of polypeptide content at the end of storage. The peptide content results are shown in Figure 3B. *P. lundensis* J8 produced the greatest peptide content, indicating the highest proteolytic capability and the largest potential for in situ spoilage. By contrast, the peptide content of chicken protein generated by *P. lundensis* J7, *A. hydrophila* Q20, *A. media* Q23, *B. thermosphacta* R1, and *B. thermosphacta* R9 were less than that of *P. lundensis* J8, suggesting diminishing spoilage potential on chicken. These findings are in accordance with the results of Fang et al., who studied the proteolytic activities of *P. lundensis* and *B. thermosphacta* on refrigerated beef and indicated that the concentration of TCA-soluble peptides generated from *P. lundensis* was the highest [47].

### 3.6. E−nose Response Analysis

**Figure 4:**
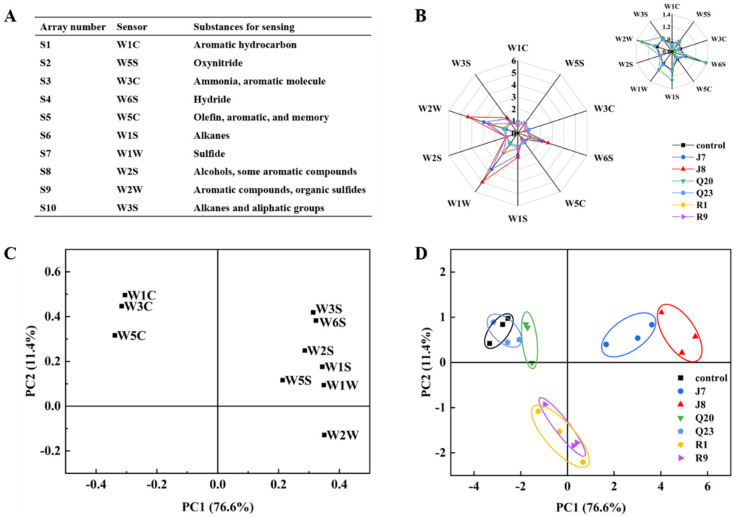
Effects of different spoilage bacteria on E-nose responses to chicken samples at the end of storage. Response substances of each E−nose sensor (**A**), radar plot (**B**), loading plot (**C**), and principle component analysis (**D**) of chicken samples incubated with different bacteria.

The E-nose is an odor-sensing array simulating biological olfaction, which is sensitive to the volatile compounds in samples [23,48]. As described in Figure 4A, 10 different sensors are found in the E-nose system, and each sensor has its sensing substances. The chicken samples were characterized by E-nose sensors to distinguish the samples incubated with different bacteria at 10 °C. As shown in the radar diagram (Figure 4B), the W1W, W2W, and W6S sensors had the strongest response intensity to volatile compounds in the chicken samples, especially the W1W and W2W sensors, indicating the abundance of sulfide, aromatic compounds, organic sulfides, and hydride in the chilled chicken samples, which was closely related to the bacteria’s metabolism. Similarly, Wang et al. [2] found that volatile compounds generated from refrigerated pork were closely associated with spoilage bacteria growth and metabolism.

Subsequently, PCA analysis was conducted for further distinction of the chicken samples contaminated with different spoilage bacteria. The loading plot (Figure 4C) shows that the first two principal components contributed approximately 88% of the total variance with PC1 and PC2 of 76.6% and 11.4%, respectively, which were sufficient to explain a large fraction of variable information in the dataset. In addition, Figure 4C also shows the relationship between E-nose sensors and principle components. W1W and W2W showed the greatest contribution to PC1, while W1C contributed the greatest to PC2, indicating that an increased PC1 value represented enhanced spoilage level. Furthermore, the two-dimensional projection of the chicken samples is shown in Figure 4D, with PC1 and PC2 as loading factors. In the score plot, the PC1 value increased in the following order: control < Q23 < Q20 < R1 < R9 < J7 < J8. The chicken samples contaminated with *Pseudomonas* spp., *Aeromonas* spp., and *Brochothrix* spp. were roughly located in three relatively independent regions, while the spatial regions of chicken sample groups incubated with the same bacteria genus were close to each other, indicating that the odor characteristics of different spoilage bacteria genera were distinctly different, and different bacteria in the same genus had similar spoilage flavors. In addition, the spatial distance between *Pseudomonas* spp. and other species was the largest, which might be related to the strong spoilage potential of *P. lundensis* [31]. Therefore, the E-nose could be applied as a promising method for making a distinction among the spoilage flavors produced by different bacteria.

### 3.7. Correlation Analysis of Spoilage Indexes and E-Nose Responses

**Figure 5:**
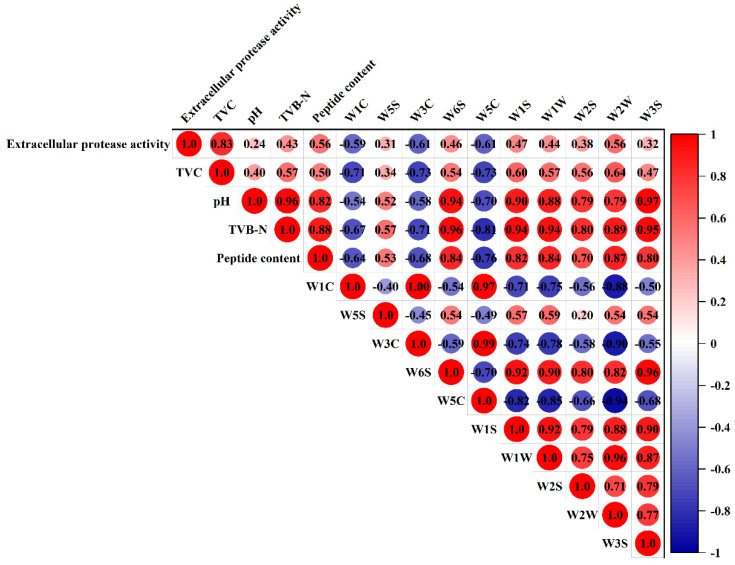
Correlation analysis among extracellular protease, TVC, pH, TVB−N, peptide content, and 10 E-nose volatile odor sensors.

The relationships among spoilage characteristics were investigated in this study by heatmap (Figure 5). The Pearson correlation coefficient (r) was applied to determine the strength of correlation (strong correlation: |r| > 0.7, weak correlation 0.3 < |r| < 0.7) [49]. The results of correlation indicated that extracellular protease activity was positively correlated with the most in situ spoilage characteristics while negatively correlated with aromatic sensors (W1C, W3C, and W5C), exhibiting that chilled chicken spoilage would lead to a loss of the pleasant odor of fresh chicken. The in situ spoilage characteristics displayed a strong positive correlation except for TVC, illustrating that the TVC of different strains during storage has no direct effect on chicken spoilage [47]. Particularly, pH, TVB-N, and peptide content also showed a strong negative correlation with aromatic sensors. Correspondingly, of the 10 E-nose sensors, W1C, W3C, and W5C were negatively correlated with the other sensors, especially W1S, W1W, and W2W. In addition, W6S, W1S, W1W, W2S, W2W, and W3S exhibited a strong positive correlation. The characteristic odor represented by the sensor indicated that spoiled chicken would produce hydride, sulfide, alcohols, organic sulfides, and other unsound odors, which would rise with the increase in storage duration and gradually replace the original aroma of fresh chicken [50].

Previous studies mainly focused on describing the abundance and diversity of spoilage microorganisms in chilled meats, while ignoring that the spoilage characteristics of bacteria could be significantly different. Herein, the spoilage potential of bacteria were evaluated based on proteolytic activities in vitro and microbial growth, biochemical changes, and volatile odor in situ. Our findings suggested that different microbial species exhibited different spoilage potential, even different isolates in the same genus. For spoilage potential analysis on meat, *P. lundensis* J8 exhibits high spoilage potential, which should be paid more attention.

## 4. Conclusions

In this study, the spoilage potential diversity of bacteria isolated from chilled chicken was studied by in vitro and in situ spoilage characterization. In vitro, *P. lundensis* J8 exhibited the strongest proteolytic activity on skim milk plates; meanwhile, it had the highest value of TVC (9.71 ± 0.04 log CFU/g), pH (6.50 ± 0.02), TVB-N (51.42 ± 0.94 mg/100 g), and peptide content (16.57 ± 0.18 mg/g) in situ at the end of storage. Compared with *P. lundensis* J8, *A. hydrophila* Q20 also presented strong proteolytic activity in vitro, whereas it exhibited the lowest growth rate on chicken and resulted in nonsignificant pH changes of chicken, which is likely the main reason for its weaker spoilage ability in situ. Therefore, it is likely that *P. lundensis* J8 plays a significant role in chilled chicken spoilage. Nevertheless, due to the fact that the present study of spoilage characterization was performed under pure culture conditions, it is necessary to further investigate the spoilage capability of different bacteria in mixed cultures in the future.

## Figures and Tables

**Table 1:** Skim milk agar assay of the bacterial isolates.

Genera	Species	Number of Isolates	Isolates	Proteolytic Activity
*Pseudomonas* spp.	*P. gessardii*	1	J1	++++
	*P. putida*	1	J4	No proteolytic activity
	*P. libanensis*	1	J5	+++
	*P. fluorescens*	1	J6	+++
	*P. lundensis*	2	J7	++
			J8	+++++
	*P. psychrophile*	1	J9	+++
	*P. azotoformans*	2	J10 and J11	++++
	*P. poae*	2	J13 and J14	+++
*Aeromonas* spp.	*A. media*	5	Q15	+++
			Q18, Q23, and Q26	+
			Q22	++
	*A. salmonicida*	2	Q16 and Q19	+++
	*A. rivipollensis*	2	Q17	+
			Q25	++
	*A. hydrophila*	2	Q20 and Q21	++++
	*A. caviae*	1	Q24	+
	*A. popoffii*	1	Q27	++++
*Psychrobacter* spp.	*P. pulmonis*	1	F28	No proteolytic activity
*Cronobacter* spp.		1	B29	+
*Escherichia* spp.		1	D30	+++++
*Brochothrix* spp.	*B. thermosphacta*	9	R1 and R3	++
			R2, R6, R7, R8, R9, R10, and R11	+++

Proteolytic activity was described by DZD/BCD results of different strains. “+”: 1.0 < DZD/BCD ≤ 1.5, “++”: 1.5 < DZD/BCD ≤ 2.0, “+++”: 2.0 < DZD/BCD ≤ 2.5, “++++”: 2.5 < DZD/BCD ≤ 3.0, and “+++++”: 3.0 < DZD/BCD.

## Data Availability

The data is included in this article.
